# A 4q35.2 subtelomeric deletion identified in a screen of patients with co-morbid psychiatric illness and mental retardation

**DOI:** 10.1186/1471-2350-5-21

**Published:** 2004-08-13

**Authors:** Ben S Pickard, Edward J Hollox, M Pat Malloy, David J Porteous, Douglas HR Blackwood, John AL Armour, Walter J Muir

**Affiliations:** 1 Medical Genetics, Molecular Medicine Centre, Univ. of Edinburgh, Western General Hospital, Crewe Road, Edinburgh, EH4 2XU, UK; 2 Psychiatry, Univ. of Edinburgh, Royal Edinburgh Hospital, Morningside Park, Edinburgh, EH10 5HF, UK; 3 Institute of Genetics, Univ. of Nottingham, Queen's Medical Centre, Nottingham, NG7 2UH, UK

## Abstract

**Background:**

Cryptic structural abnormalities within the subtelomeric regions of chromosomes have been the focus of much recent research because of their discovery in a percentage of people with mental retardation (UK terminology: learning disability). These studies focused on subjects (largely children) with various severities of intellectual impairment with or without additional physical clinical features such as dysmorphisms. However it is well established that prevalence of schizophrenia is around three times greater in those with mild mental retardation. The rates of bipolar disorder and major depressive disorder have also been reported as increased in people with mental retardation. We describe here a screen for telomeric abnormalities in a cohort of 69 patients in which mental retardation co-exists with severe psychiatric illness.

**Methods:**

We have applied two techniques, subtelomeric fluorescence in situ hybridisation (FISH) and multiplex amplifiable probe hybridisation (MAPH) to detect abnormalities in the patient group.

**Results:**

A subtelomeric deletion was discovered involving loss of 4q in a patient with co-morbid schizoaffective disorder and mental retardation.

**Conclusion:**

The precise region of loss has been defined allowing us to identify genes that may contribute to the clinical phenotype through hemizygosity. Interestingly, the region of 4q loss exactly matches that linked to bipolar affective disorder in a large multiply affected Australian kindred.

## Background

The isolation of unique DNA probes from the sub-telomeric regions of all chromosomes has opened up a field of cytogenetics research that was previously inaccessible to conventional karyotyping protocols [1]. Since then a number of studies have shown that cryptic structural abnormalities (deletions, duplications etc.) in the subtelomeric regions are relatively commonly found in groups of individuals with idiopathic mental retardation (UK; learning disability; LD). The biological attributes of these chromosomal regions may explain this interesting link. The frequency of meiotic recombination is at its highest at the ends of chromosomes (recently confirmed in the Icelandic microsatellite map of the human genome [2]). Therefore errors in this process should randomly result in a greater frequency of unbalanced chromosome rearrangement products at telomeres. There also appears to be a greater density of genes at the ends of some chromosomes, especially those with non-staining R-bands. Thus, any telomeric copy number change is likely to affect several genes; potentially resulting in clinical features typical of a contiguous gene syndrome – dysmorphisms, developmental delay and mental retardation. A number of reports have now shown that 0.5%–23% of idiopathic mental retardation cases are associated with cryptic translocations in the vicinity of chromosome telomere (see [3-17] and [18] for a recent review).

FISH, using a commercially available set of subtelomeric probes is the most commonly used screening technique [19,20]. Variations on the theme of FISH (e.g. SKY and CGH) have also been employed. More recently, methods that rely on the detection of copy number changes at subtelomeric loci have been described. MAPH [21-24] is one such technique in which probes are representatively amplified by the polymerase chain reaction following hybridisation to a patient's genomic DNA sample to generate a quantitative profile of subtelomeric sequence copy number.

Psychiatric disorders such as schizophrenia (SCZ) and bipolar affective disorder (BPAD) are relatively common in the general population and there is much evidence for a genetic component to susceptibility (for a review see [25]). However, it is clear from the lack of consistent findings from linkage mapping and association studies that they are likely to be complex and aetiologically heterogeneous disorders. For example, several genes might act simultaneously (oligogenic action) or interact (epistasis) to produce the clinical phenotype in any individual, and those genes might be different in different individuals (locus heterogeneity). An alternative to cohort based linkage and association approaches uses cytogenetic abnormalities as direct pointers to candidate gene loci and this has been successfully applied to patients with psychiatric disorders resulting in the identification of a number of candidate susceptibility genes including *DISC1/DISC2 *[26], *DIBD1 *[27] and *GRIA3 *[28]. The chromosome abnormalities that disrupted these genes were reciprocal translocations visible by standard cytogenetic methods.

The risk of schizophrenia and affective disorders in patients with idiopathic mild mental retardation is significantly raised and it is well established that schizophrenia is three times more common in this group than the general population and that there is a strong familial element [29]. Both bipolar illness and major depressive disorder have also been described as of increased prevalence in the population with mild mental retardation. The study also revealed a previously undetected complex re-arrangement between chromosomes 2 and 11, and a case of trisomy X, but did not address subtelomeric changes. It strongly suggested however that the co-association between mental retardation and schizophrenia is highly familial with greater rates of both schizophrenia and co-morbid schizophrenia/mental retardation occurring in the families of co-morbid probands compared to families of probands with schizophrenia alone or with mental retardation alone. Limbic system (amygdalo-hippocampal) neuropathology is especially pronounced in this group [30]. We have formed the hypothesis that patients who are co-morbid for severe psychiatric illness and mental retardation may be homogenous in their pathophysiology and that, in addition to large-scale structural chromosomal abnormalities, they may harbour as yet undetected cryptic telomeric changes. To test this we have screened a series of 69 patients co-morbid for mental retardation and psychiatric illness using fluorescence in situ hybridisation (FISH) and multiplex amplifiable probe hybridisation (MAPH).

## Methods

### Patient Cohort

Local research ethics permission was obtained for this study. The patients were initially ascertained through computerised psychiatric clinical case-registers that allowed us to identify adults with dual diagnosis of psychosis and mental retardation. A specific psychiatry service exists in Scotland to meet the needs of patients with mental retardation who also suffer from psychiatric disorders and initial clinical diagnoses were confirmed by consultation between the relevant specialist clinician involved and the research team member who is also a specialist in the psychiatry of mental retardation (WM). Confirmation that IQ fell within the mild range of mental retardation was obtained from case records.

69 patients with mild mental retardation (IQ 70 to around 50) and a referral diagnosis of co-existing schizophrenia or major affective disorder were studied. Parental samples were not available in many cases due to the age of the probands. This cohort is a subset of 74 originally ascertained subjects: 5 were removed because of aneuploidy (2 cases of 47(XXX)) or after more thorough psychiatric evaluation. One discounted subject with only mental retardation possessed a 6q subtelomeric deletion as determined by several MAPH probes (data not shown). The lifetime version of the Schedule for Affective Disorders and Schizophrenia (SADS-L [31]) along with extensive case record review, and interviews with key carers and relatives was used to gather the information needed to make a diagnosis of schizophrenia or affective disorder according to the Diagnostic and Statistical Manual 4^th ^Edition (DSM-IV [32]). Diagnosis was finalised by consensus between two experienced psychiatrists (DB, WM) one of whom specialises in the psychiatry of mental retardation (WM). SADS-L has previously been successfully used in people with mild mental retardation [29] to establish psychiatric diagnoses.

Overall 49 subjects met the DSM-IV criteria for definite schizophrenia, 3 for schizoaffective disorder, 11 for Bipolar I Disorder, 1 for recurrent Major Depressive Disorder (unipolar depression). In addition, 5 subjects were diagnosed as having a unspecified functional psychosis (DSM-IV 298.9, Psychotic disorder NOS).

None had co-existing Down Syndrome or Fragile X disorder. A breakdown of the patients into their clinical categories and methodology of screening is presented in table 1.

**Table 1:** Subject classification and analysis Breakdown of subjects into their diagnostic categories and applied experimental methodology. MR; mental retardation, SCZ; schizophrenia, BP1; bipolar affective disorder I, SCAFF; schizoaffective disorder, UFP; unspecified functional psychosis, UPR; unipolar depression.

**Clinical category**	**MAPH alone**	**MAPH and FISH**	**FISH alone**
MR/SCZ	34	7	8
MR/BP1	6	3	2
MR/SCAFF	1	2	0
MR/UFP	3	0	2
MR/UPR	1	0	0
TOTAL SCREEN	45	12	12

### DNA extraction

DNA was extracted from venous blood samples (10 mls) of all patients by standard methods using Nucleon BACC2 kits (Nucleon Biosciences). 1 mg/ml dilutions were prepared for MAPH.

### MAPH

All 57 MAPH samples were tested in triplicate using the subtelomeric screening set described previously [23]. All samples were anonymised prior to MAPH analysis. Each sample was tested three times, and putative positives identified by a univariate method (standard hypothesis testing against a normal distribution) and multivariate methods employed by the software SYSTAT 8.0 (Bivariate scattergraphs and Hadi outlier analysis). Four putative positives were divided into one confident (univariate analysis, p < 0.01, corrected for multiple observations) and three possible (univariate analysis, p < 0.05, corrected for multiple observations, Hadi outlier distance >4, all three results reporting a consistent change: either all >1.0 or <1.0) positive results. The three "possible" positives have since been discounted since they involved gain of the 20p telomeric probe ST18E1, which from experience with normal control subjects has shown to have unacceptably high measurement error. This probe has been replaced in more recent formulations of the subtelomeric probe set.

### Subtelomeric FISH

Blood samples from a subset of the patients were cultured in Peripheral Blood Medium (Sigma) for 72 hours. After colcemid treatment for one hour, lymphocytes were lysed and fixed in methanol:acetic acid (3:1). Fixed metaphase material was dropped onto microscope slides. Each slide was hybridised by three fluorescently labelled probe mixes (ToTelVysion, Vysis Inc.) under separate coverslips. All 15 mixes covering every subtelomeric region for a patient could thus be analysed on 5 slides. Images were captured on a Zeiss Axioskop2 microscope coupled to a Macintosh G4 computer running SmartCapture2.1 software (Digital Scientific). Five metaphases were scored for each probe mix.

## Results

### A subtelomeric deletion identified in one subject

Complete accord was seen in the 12 instances where both screening methodologies were used. The FISH approach did not detect any subtelomeric abnormalities (including balanced translocations, which would not be observable by MAPH). However, MAPH identified a subject with a loss of one copy of the 4q subtelomeric region (p < 1 × 10^-3^, corrected for multiple observations). It is unlikely that this copy number change (4q^-^) defined by MAPH represents an irrelevant polymorphism; no similar change was found on analysis of 83 unrelated control individuals, giving an upper (95% confidence) limit of 1.6% for the frequency of this variant.

### Precise definition of 4q loss

**Figure 1:**
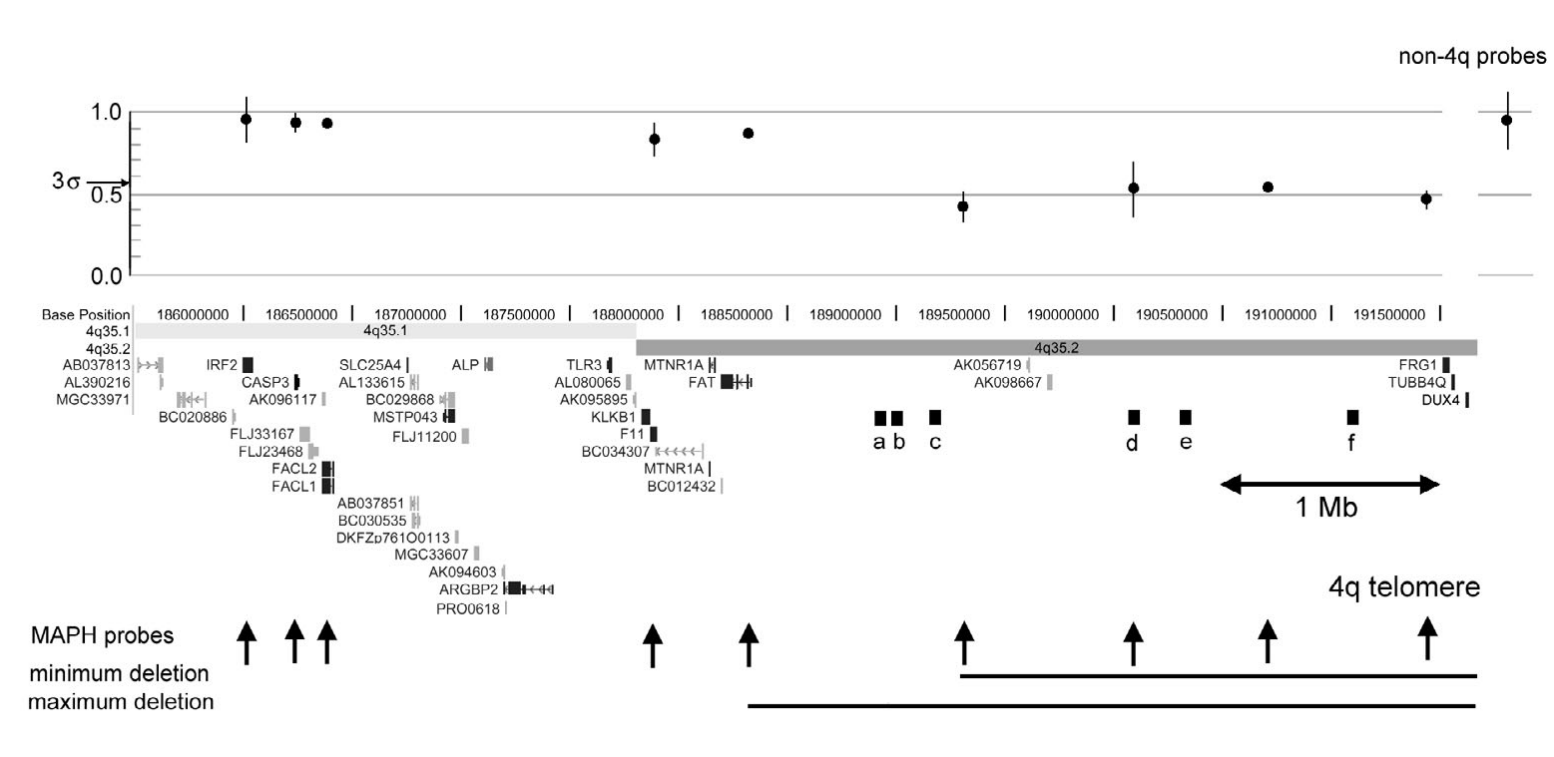
**Subtelomeric region of chromosome 4q **Only annotated, unique chromosome sequence is shown, derived from the November 2002 version of the UCSC Human Genome assembly (subtelomeric repeats would extend to the right of the diagram). A scale bar and the gene content (see Table 2) of the region are shown. The positions of the MAPH markers are also shown which allowed the maximum and minimum extents of the deletion to be defined (black bar). Above the chromosome region is shown the result of duplicate analysis from each MAPH probe (mean +/- 95% CI), together with the 3 standard deviation threshold and the results from the other control probes (mean +/- 95% CI).

Additional MAPH probes were designed to determine the extent of the 4q deletion (fig. 1). The results show that the proximal boundary of the subtelomeric deletion is between the *FAT *gene and the proximal end of clone 713c19 (Genbank accession number AC108073). P values for boundary probes (Ho, value = 1.00), p < 5 × 10^-5 ^for deletion and p > 0.05 for normal dosage. This 4q deletion encompasses a region of annotated genomic DNA of approximately 3 Mb. The transcript map of this region is not yet completely defined (see Table 2) but contains at least 10 transcriptional units with varying levels of authenticity/experimental evidence attached to each one.

**Table 2:** Gene content of 4q deletion Genes/putative transcriptional units within the deleted region on chromosomes 4q. ESTs a-f are represented in figure 1. An attempt to gauge the approximate expression levels of each gene was based on the number of EST clones present in the UCSC Human Genome Browser (Nov.2002/Apr.2003 releases). A brief summary of gene function and a representative accession number, where informative, is also included. TUBB4Q (4q35) is omitted from this list because it is a confirmed pseudogene.

**4q35 GENE**	**EST exp.**	**Function/comments**
*FAT*	++++	Cadherin-related tumor suppressor homologue precursor
*EST a*	+	(BE856720) Novel.
*EST b*	+	(BM806339) Novel. Contains 5 1/2 copies of 34aa repeat motif
*EST c*	++	(AI917275) Novel. No obvious ORF
*ZFP42/FLJ32157*	+	(AK056719) Similar to transcriptional repressor protein YY1
*FLJ25801*	+	(AK098667) Protein contains SMC (chromosome segregation ATPase) domain and PRY/SPRY domains (unknown function).
*EST d*	+++	(BU571187) Novel.
*EST e*	+	(BC033535) Novel.
*EST f*	+	(BC029568) LOC256307 novel predicted gene
*FRG1*	++++	Facioscapulohumeral muscular dystrophy region gene 1
*DUX4*	+	Homeobox protein, multiple copies.

## Discussion

Unlike simple chromosomal translocations, large deletions associated with a certain condition can present many candidate genes for further study. In the context of psychiatric disorders velo-cardio-facial syndrome (VCFS or del22q11 syndrome) offers a model of how a cryptic deletion associated with schizophrenia has highlighted several candidate genes for future study [33-35].

We used two methods to screen for subtelomeric changes in our cohort of patients. The MAPH technique has proved a fast and accurate method for determining copy number changes in the human genome and represents a cost-effective route to the screening of large numbers of patients. In addition, the disorders that can be studied by this approach are limited only by the design of suitable primers so that screening for both single and contiguous gene disorders is feasible. MAPH has also been proved to be a simple method to map deletion breakpoints with greater resolution than FISH. The subtelomeric FISH approach is a more demanding approach because of the patient sample preparation, the requirement for specialised microscopy equipment, the cost of the commercial probe sets and the labour involved. Nevertheless, FISH has some advantages: the FISH approach is the only way to detect balanced chromosome rearrangements such as inversions and translocations. Other studies of subtelomeric regions in mental retardation subjects have identified such chromosomal aberrations. In addition, FISH has been a vital technique for identifying disrupted genes such as *DISC1 *in psychiatric patients with (non-subtelomeric) chromosomal rearrangements. Therefore, the selection of the screening technique should be determined by the number of cases to be studied and the nature of the abnormalities expected.

We have identified a subtelomeric deletion within our cohort of 69 patients using the MAPH and FISH methodologies. This 4q deletion is associated with a co-morbid phenotype of schizoaffective disorder and mild mental retardation (fullscale IQ between 60 and 70). Consent was not forthcoming to determine whether the deletion was of parental or *de novo *origin. However, psychiatric illness has not been diagnosed in other members of the family. We cannot, therefore, formally link the presence of the deletion with mental retardation and/or psychiatric illness in the patient. The annotation of transcripts at 4q35.2 is currently an active area of research (see fig. 1 and table 2) because of good linkage evidence (LOD score of 3.2 for microsatellite marker D4S1652) from an extended Australian kindred multiply affected with bipolar affective disorder [36-38]. Importantly, the principal linkage region almost exactly matches the deletion interval observed in our patient. The 4q deletion patient has been diagnosed with schizoaffective disorder (DSM-IV 295.7) – with periods of psychotic depression but also mood incongruent hallucinations and delusions. This is in contrast to the clear bipolar affective disorder diagnosed for members of the described linkage family. However, it has been repeatedly observed that schizoaffective disorders and bipolar affective disorders overlap clinically and are indeed often difficult to separate. One postulated explanation for the now frequently reported linkage overlaps between bipolar illness, schizoaffective disorders and schizophrenia is that the inherited susceptibility is for psychosis rather than a specific disorder (reviewed in [39]).

The subtelomeric region of 4q is also interesting because it contains a candidate gene, *FRG1*, for facioscapulohumeral muscular dystrophy. The 4q patient does not show any of the typical features of this disorder but this can be explained by the fact that copy number does not appear to be critical for the onset of the disorder [40]. Rather, the proximity of the gene to a variable number telomeric repeat sequence (D4Z4) seems to be the chief determinant of pathology [41].

Of the 69 patients with clear co-morbidity, one (1.4%) possessed a single copy subtelomeric deletion. This frequency is in line with those from studies of individuals with mental retardation alone [18]. As more studies examine chromosomal integrity in people with mental retardation or other conditions we hope that replication of subtelomeric abnormalities will be observed, perhaps leading to the eventual clinical definition of range of 'subtelomeric syndromes' such as that recently described for the subtelomeric deletion of 1q [42].

## Conclusions

The identification of the precisely delimited 4q deletion may contribute to the mapping of the susceptibility gene for psychiatric illness at this locus. The finding of bipolar affective disorder linkage to this region suggests that, in this case at least, the schizoaffective and mental retardation components to the co-morbid phenotype may be discrete and genetically separable in the manner of other contiguous gene disorders. If it is assumed that either component of the clinical phenotype is caused by haploinsufficiency then examining the comparative expression levels of candidate genes in normal, bipolar-linked and 4q deleted lymphoblastoid cell lines might provide a quick route to the identification of causative genes. Alternatively, gene association studies may be required to identify the candidate psychiatric illness gene at 4q35. The high rate of recombination in subtelomeric DNA means that a higher density of genetic markers will be required to establish linkage or association reliably; conversely, once detected, the high rate of recombination will allow high resolution fine mapping of significant loci.

## Competing interests

None declared.

## Authors' contributions

BSP participated in the design of the study and the FISH analysis and drafted the manuscript. EJH and JALA carried out all MAPH assays and associated statistical analyses. MPM carried out blood culture and FISH analysis. WJM conceived the study and was responsible for the generation of all clinical data, and DHRB and DJP participated in the study design and coordination. All authors read and approved the final manuscript.

## Pre-publication history

The pre-publication history for this paper can be accessed here:


